# Oral care practices in stroke: findings from the UK and Australia

**DOI:** 10.1186/s12912-021-00642-y

**Published:** 2021-09-15

**Authors:** Munirah Bangee, Cintia Mayel Martinez-Garduno, Marian C. Brady, Dominique A. Cadilhac, Simeon Dale, Margaret A. Hurley, Elizabeth McInnes, Sandy Middleton, Tahera Patel, Caroline L. Watkins, Elizabeth Lightbody

**Affiliations:** 1 Faculty of Health and Care, University of Central Lancashire, Preston, PR1 2HE UK; 2 St Vincent’s Health Network Sydney, St Vincent’s Hospital Melbourne & Australian Catholic University, Sydney, Australia; 3 Nursing, Midwifery and Allied Health Professions Research Unit, Glasgow Caledonian University, Glasgow, UK; 4 School of Clinical Sciences at Monash Health, Monash University, VIC Melbourne, Australia

**Keywords:** Nursing, Oral hygiene, Mouth care, Protocol, Survey, Stroke

## Abstract

**Aims:**

To examine current practice, perceptions of healthcare professionals and factors affecting provision for oral care post-stroke in the UK and Australia.

**Background:**

Poor oral care has negative health consequences for people post-stroke. Little is known about oral care practice in hospital for people post-stroke and factors affecting provision in different countries.

**Design:**

A cross-sectional survey.

**Methods:**

Questionnaires were mailed to stroke specialist nurses in UK and Australian hospitals providing inpatient acute or rehabilitation care post-stroke. The survey was conducted between April and November 2019. Non-respondents were contacted up to five times.

**Results:**

Completed questionnaires were received from 150/174 (86%) hospitals in the UK, and 120/162 (74%) in Australia. A total of 52% of UK hospitals and 30% of Australian hospitals reported having a general oral care protocol, with 53% of UK and only 13% of Australian hospitals reporting using oral care assessment tools. Of those using oral care assessment tools, 50% of UK and 38% of Australian hospitals used local hospital-specific tools. Oral care assessments were undertaken on admission in 73% of UK and 57% of Australian hospitals. Staff had received oral care training in the last year in 55% of UK and 30% of Australian hospitals. Inadequate training and education on oral care for pre-registration nurses were reported by 63% of UK and 53% of Australian respondents.

**Conclusion:**

Unacceptable variability exists in oral care practices in hospital stroke care settings. Oral care could be improved by increasing training, performing individual assessments on admission, and using standardised assessment tools and protocols to guide high quality care. The study highlights the need for incorporating staff training and the use of oral care standardised assessments and protocols in stroke care in order to improve patient outcomes.

**Supplementary Information:**

The online version contains supplementary material available at 10.1186/s12912-021-00642-y.

## Background

Oral care is essential for optimal oral health and includes activities such as healthy eating, drinking and tooth brushing [1]. Physical and cognitive difficulties, reduced conscious level and other co-morbidities, increase the risk of poor oral health post-stroke [2] and make it challenging for a person to undertake independent oral care. People post-stroke need oral care support from others, but if it is not managed appropriately, it can negatively affect physiological, social and psychological wellbeing, and can lead to discomfort, toothache, periodontal disease and pneumonia [3, 4]. Inpatient mortality is nearly six times higher in those patients with pneumonia [5] and the risk of developing pneumonia could be reduced by systematic oral hygiene care [6]. Ultimately, these problems impede adequate nutritional intake, prolong hospital stay and impact recovery [3].

Unfortunately, despite the known consequences, post-stroke oral care is often neglected, particularly for those who have functional and cognitive difficulties where oral care is often undertaken by nurses or carers/family members [7]. The important role nurses and carers perform is highlighted in UK clinical guidelines, which recommend that staff and carers of people post-stroke should be trained in the assessment and management of oral hygiene [8]. These recommendations are reiterated in Australian guidelines [9] and New Zealand guidelines [10]. Further to this, both the UK and Australian guidelines suggest the use of cleaning agents and equipment in oral care post-stroke. In contrast, Canadian Stroke Best Practice Recommendations suggest that people post-stroke should have an assessment and care protocol [11].

Despite the importance of staff training outlined in clinical guidelines, there is little empirical evidence regarding staff knowledge on delivering this care. There have been two qualitative studies exploring the perceptions of healthcare professionals [12], as well as the perceptions of stroke survivors and their carers of oral care post-stroke [13]. These studies reported that staff felt insufficiently trained to deliver oral care effectively [12, 13]. In addition, three surveys on in-patient oral care practice post-stroke (Scotland, England and Malaysia) reported variability in oral care practices [13–15].

Oral care may vary in clinical practice due to the lack of high quality evidence to underpin the management of, and interventions for, people after stroke [16, 17]. Overall, there remains a lack of knowledge about the type of oral care currently provided in hospital post-stroke, as well as the factors associated with providing adequate oral care. Obtaining an in-depth understanding of the attitudes and knowledge-base of nursing staff, including barriers to oral care, is important so that oral care practices post-stroke can be improved.

The aims of this study were to 1) identify current practices of oral care post-stroke, 2) explore perceptions of healthcare professionals on their practice of oral care post-stroke and, 3) to identify the barriers and enablers to providing oral care in hospital post-stroke in the UK and an international comparator, Australia.

## Methods

### Design

A cross-sectional survey to explore the oral care practices post-stroke in the UK and Australia. Australia was selected as the international comparator since it has comparable healthcare systems to the UK. Both countries have similar health care performance as well as similar mortality rates following an ischemic stroke [18]. The survey was carried out in accordance with the research governance regulations in each country and the results are reported using the STROBE checklist.

### Hospital selection

All hospitals known to provide stroke services (including stroke rehabilitation) in the UK and Australia were contacted. For the UK, hospitals in England, Wales and Northern Ireland were identified via the Roayal College of Physicians' Sentinel Stroke National Audit Programme (SSNAP). Hospitals in Scotland were identified via the Scottish Stroke Care Audit. For Australia, hospitals were identified from the Stroke Foundation Organisational Survey [19] and the Stroke Foundation’s National Stroke Audit - Rehabilitation Services Report 2016 [20]. 

### Data collection

Data were collected from April to November 2019 using a self-administered postal questionnaire which took approximately 20 minute to complete.

#### Questionnaire content and development

A 20 minute purposefully designed questionnaire was developed, informed by a recent literature review and a previous UK study [15]. The questionnaire was piloted with an expert panel of stroke clinicians (4 from the UK and 4 from Australia) to review the questions, response options and to determine completion time. The questionnaire (Appendix S1) comprised six sections that included the following topics: (1) respondent's demographics, (2) hospital and stroke service characteristics, (3) oral care practices, (4) assessment of oral care, (5) oral care resources and equipment available and (6) factors influencing the provision of oral care. All questions were closed but a free text option was available for some questions, where the respondent could write in an alternative answer to the choices given. These responses were collated and either re-categorised into the original categories where appropriate or into new categories. For the majority of questions in section 4, respondents completed a six Likert-type scale (highly likely, likely, unsure, unlikely,highly unlikely, not applicable). For section 6, respondents indicated their level of agreement on a five-point Likert scale (strongly agree, agree, unsure, disagree, strongly disagree).

#### Questionnaire distribution

One key participant was identified from the acute stroke service and/or from the rehabilitation service (if any) at each hospital. Target participants were generally the stroke unit coordinator or stroke-specialist nurse, but may have included the stroke unit nurse, unit manager or the clinical lead. Once these key contacts were identified at each hospital, an advance e-mail was sent the day before the questionnaire distribution to notify potential participants of the upcoming survey. Participation was voluntary and informed consent was implied after completion and return of the questionnaire which was made explicit in the Participant Information Statement and approved by all ethics committees, no survey participants were aged under 18 years. Questionnaires were posted with a reply-paid envelope. Participants had the option to return completed questionnaires via post or electronically by scanning and e-mailing it back to the investigators. Non-respondents were contacted up to five times, initially at 3 weeks and every 2 weeks thereafter (three times by e-mail and twice by telephone) to optimise response rates. Non-respondents were given an additional electronic copy of the questionnaire sent as an attachment via e-mail at each follow up.

### Data analysis

Data were analysed using descriptive statistics and reported as counts and percentages. UK and Australian responses were analysed separately. There were three levels of non-response to the survey; firstly, the hospitals which declined to participate at the outset, secondly, the hospitals which agreed to participate but which then, subsequently, did not return a questionnaire and thirdly, non-response to specific questions within a returned questionnaire. No adjustment in the analyses were made for the first two types of non-response because it was assumed that responding hospitals were representative of the wider hospital group. Where non-response occurred for specific questions in the returned questionnaires, the counts and percentages for non-response are shown in all tables and figures. For the purpose of data analysis, the categories highly likely and likely were combined to create a single likely category; unlikely and highly unlikely were combined into an unlikely category.

Data were entered in REDCap electronic data capture tools [21, 22] and prepared for statistical analysis using SPSS (IBM SPSS Statistics 26, IBM Corporation, Armonk, NY, USA).

## Results

### Respondents and hospital characteristics

In the UK, 261 participants from eligible hospitals were initially contacted to take part in the survey. Of these, 87 declined to participate or did not respond to the invitation to participate. Therefore, 174 participants were sent the questionnaires and 150 (86%) were completed and returned. In Australia, 172 participants from eligible hospitals were initially contacted to take part and were sent a questionnaire, of these 10 immediately declined and of the remaining 162, 120 (74%) completed and returned the questionnaire.

The majority of respondents were nurses; 77% in the UK and 85% in Australia. A total of 79% of UK and 73% of Australian respondents had a stroke-specific role within the service. The majority of respondents were females; 86% in the UK and 92% in Australia. A total of 28% UK and 30% Australian respondents were between the ages of 41 and 50 years. Respondents generally worked within an acute stroke unit. Table 1 shows key stroke service demographics.

**Table 1:** Key stroke service demographics

	UK n (%)	Australia n (%)
**Hospital unit**		
Acute stroke unit	70 (47)	53 (44)
Ward with stroke beds	9 (6.0)	10 (8.3)
Integrated unit	48 (32)	17 (14)
Rehabilitation unit	21 (14)	35 (30)
Other	2 (1.3)	4 (3.3)
Not reported	0 (0.0)	1 (0.8)
**Hospital setting**		
Tertiary	60 (40)	55 (46)
Non-Tertiary with Emergency Department	67 (45)	47 (39)
Non-Tertiary without Emergency Department	20 (13)	12 (10)
Other	1 (0.7)	5 (4.2)
Not reported	2 (1.3)	1 (0.8)
**Stroke service availability**^**a**^		
Thrombolysis	124 (83)	84 (70)
Endovascular therapy	54 (36)	57 (48)
Neurovascular imaging	120 (80)	88 (73)
Telemedicine	81 (54)	65 (54)
Rehabilitation	130 (87)	78 (65)
Neurosurgery	55 (37)	56 (47)

^a^More than one option permissible

### Current practices of oral care post-stroke

A total of 52% of responding hospitals in the UK and 30% in Australia had a general oral care protocol or guideline for all patients. However, only 17% of UK and 5.8% of Australian responding hospitals had protocols specifically for people with stroke. Of those having general and stroke-specific protocols, 83% in the UK and 70% in Australia reported that clinical staff were likely to use them in clinical practice.

Staff had received oral care training in the last year in 55% (*n* = 83) of UK and 30% (*n* = 36) of Australian responding hospitals. This training was provided to registered nurses in 99% and healthcare assistants in 87% of UK hospitals. In Australia, it was provided to registered nurses in 92% and enrolled nurses in 75% of hospitals. The training was provided by speech and language therapists or dentists/dental hygienists in 42% and 14% of UK and in 64% and 11% of Australian hospitals respectively.

An oral care assessment tool was used in 53% (*n* = 80) of UK responding hospitals and 13% (*n* = 16) of Australian responding hospitals. Of those hospitals using tools, 50% of UK and 38% of Australian hospitals used hospital-specific tools. The standardised Mouth Care Assessment Tool [23] was used in 15% of UK and 31% of Australian hospitals, and the Oral Health Assessment Tool [24] was used in 14% of UK and 31% of Australian hospitals.

**Fig. 1:**
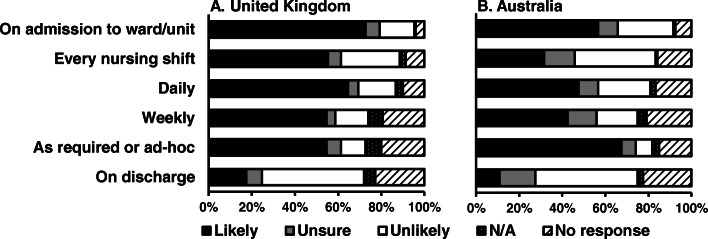
Likelihood of undertaking oral care assessments in the UK (left) and in Australia (right)

Oral care assessments were likely to be undertaken on admission to the stroke unit in 73% of UK and 57% of Australian responding hospitals. Respondents’ views on the likelihood of undertaking oral care assessments at other time-points are illustrated in Fig. 1 and Table S1. In the UK, the staff groups who were reported as likely to undertake oral care assessments were registered nurses (91% of respondents), speech and language therapists (91% of respondents) and healthcare assistants (77% of respondents). In Australia, speech and language therapists were likely to undertake assessments (96% of respondents), but fewer healthcare assistants were reported as likely to undertake assessments (33% of respondents).

The equipment available is shown in Table 2/Figure S1. For patients that did not have their own oral care equipment/products, most hospitals provided manual toothbrushes and toothpaste. In the UK, 58% of hospitals provided Corsodyl/chlorhexidine compared to 17% in Australia. Only 2.0% of UK hospitals provided sodium carbonate compared to 43% of Australian responding hospitals. Oral fluids for the management of dry mouth were available in 89% of UK and 87% of Australian hospitals.

**Table 2:** Oral care products available in UK and Australian hospitals

	UK n (%)	Australia^**a**^ n (%)
Manual toothbrush	144 (96)	104 (87)
Electric toothbrush	2 (1.3)	0 (0.0)
Suction toothbrush	38 (25)	38 (32)
Suction equipment	125 (83)	96 (80)
Denture brush	14 (9.3)	4 (3.3)
Tongue scraper	16 (11)	8 (6.7)
Foam swab	88 (59)	84 (70)
Glycerine swab	23 (15)	28 (23)
Dental floss	2 (1.3)	0 (0.0)
Soft cloth/towel	82 (55)	69 (58)
Toothpaste	145 (97)	101 (84)
Mouthwash	61 (41)	58 (48)
Mouthwash tablets	39 (26)	6 (5.0)
Denture adhesive	14 (9.3)	8 (6.7)
Steradent	46 (31)	15 (13)
Corsodyl/chlorhexidine	87 (58)	20 (17)
Sodium bicarbonate	3 (2.0)	51 (43)
Saline/sodium chloride solution	41 (27)	54 (45)
Sodium hypochlorite (Milton)	1 (0.7)	5 (4.2)
Bleach	0 (0.0)	1 (0.8)
Ascorbic acid/Vitamin C	9 (6.0)	22 (18)
Other decontaminants e.g. Antibiotic gel	11 (7.3)	9 (7.5)
Biotene/oral gel	5 (3.3)	2 (1.7)
Moutheze	4 (2.7)	0 (0.0)
Other	8 (5.3)	5 (4.2)

^a^One respondent from Australia did not answer the question

### Perceptions by healthcare professionals on their practice of oral care

**Fig. 2:**
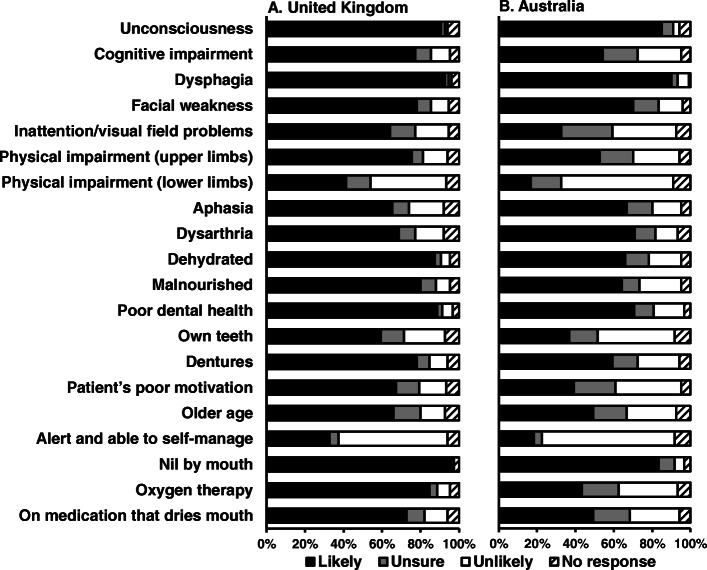
Patient factors reported to influence whether an oral care assessment was undertaken for the UK (left) and Australia (right)

Patient factors reported to influence whether an oral care assessment was undertaken are illustrated in Fig. 2 and Table S2. The most likely factor to influence the decision to undertake an assessment in the UK, was patients being nil by mouth ((95% respondents) whilst in Australia it was dysphagia (90% respondents). Patients being alert and able to self-manage was reported as the least likely factor to influence whether an assessment was undertaken (57% of UK and 69% of Australian respondents).

Registered nurses were likely to provide oral care when patients could not manage their own oral care (95% of UK and 96% of Australian respondents). Healthcare assistants/assistants in nursing were also likely to provide oral care in the UK (95%) whereas fewer respondents reported this in Australia (70%). Staff were expected to clean natural teeth and dentures twice a day (62% and 63% of UK and 56% and 45% of Australian responding hospitals respectively). Staff were expected to perform oral care for nil by mouth patients three times a day in 54% of UK and 55% of Australian hospitals.

**Fig. 3:**
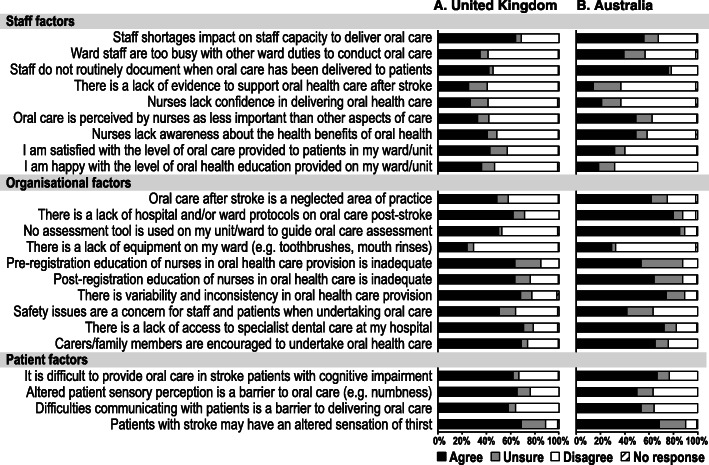
Staff factors, organisational factors and patient factors reported to influence oral care provision in the UK (left) and Australia (right)

The main staff factor to influence oral care provision in the UK was staff shortage (64% respondents), whereas in Australia it was the lack of documentation of practices (76% respondents). A total of 57% of UK and 62% of Australian respondents disagreed that nurses lacked confidence in delivering oral care. Only 43% of UK and 32% of Australian respondents were satisfied with the level of oral care provided in their hospital (see Fig. 3 and Table S3).

In general, it was agreed that oral care was a neglected area of practice (49% of UK and 62% of Australian respondents). Education and training of nurses in oral care provision was deemed inadequate in both pre-registration (63% of UK and 53% of Australian respondents) and post-registration (63% of UK and 64% of Australian respondents). A lack of protocols on oral care was also highlighted (62% of UK and 80% of Australian respondents; see Fig. 3 and Table S3).

### Barriers and enablers to providing oral care in hospital post-stroke

In terms of barriers to oral care, cognitive impairment made it difficult to provide oral care post-stroke (62% of UK and 67% Australian respondents) as did altered patient sensory perception (65% of UK and 50% of Australian respondents); see Fig. 3 and Table S3).

## Discussion

To our knowledge, this is the first concurrent survey conducted across the UK and Australia to identify and compare current practice of oral care post-stroke, as well as the factors associated with providing this care.

A significant number of hospitals in both countries did not have a protocol nor use an assessment tool on their stroke units, despite standardised assessment tools being recommended for people post-stroke [25]. Where an assessment tool was used, it was more likely to be a hospital-based tool. Our findings suggest a gradual improvement in the availability of oral care protocols post-stroke in UK hospitals when compared to previous surveys. Protocol availability in hospitals has increased from between 0 and 21% [13, 15] to 52% in the current study.

Almost half of UK and two-thirds of Australian responding hospitals did not report providing oral care training. This finding directly contradicts the National Clinical Guidelines of both countries [8, 9], which recommend that staff should be trained in the assessment and management of oral care. However, the provision of training in the UK has increased from between 0 and 33% of units [13, 15] to 55% in the current study. The availability of staff training in Australia (30%) was similar to a previous survey across Malaysia (28%) [14]. Our findings also highlighted that training was mostly provided to junior members of the nursing team such as healthcare assistants who are more likely to undertake oral care post-stroke in UK hospitals. The training available was generally provided by speech and language therapists. Dentists or dental hygienists only provided the training in a small number of hospitals.

The equipment available in hospitals varied considerably in both countries. Consistent with previous surveys [13, 15], basic equipment such as toothbrushes and toothpastes were available in most hospitals, in fact the availability of toothbrushes in UK hospitals has increased from 74% in a previous survey to 96%. The use of available rinses varied between UK and Australian settings; Corsodyl/chlorhexidine was available more in the UK than Australia, while sodium carbonate was available more in Australian settings. These variations may not be clinically important as a randomised controlled trial showed that 0.2% chlorhexidine, saline solution, and sodium bicarbonate, all kept the oral mucous membrane in good condition in critically ill patients [26]. However, there is limited evidence to guide the choice of best cleaning agents and equipment to use in oral care in stroke, as well as the use of protocols, assessment and training [17].

Despite the lack of high quality evidence on best oral care practices for people with stroke, good clinical practice recommendations are available, underpinned by evidence from small randomised controlled trials [27–29], a scoping review [7] and a Cochrane review [30]. However, our findings suggest that these recommendations are only being implemented in some hospitals. In the UK, the guidelines suggest that people with stroke should have oral care three times a day and use a suitable cleaning agent (toothpaste and/or chlorhexidine) to brush teeth and clean gums [8]. In Australia, the guidelines suggest that chlorohexidine with oral hygiene instructions, and/or assisted brushing may be used to improve patient outcomes [9]. However, chlorohexidine is only available in 17% of Australian hospitals, with most hospitals only providing basic products such manual toothbrushes and toothpaste. Both guidelines recommend that people with stroke, staff and carers should be educated in oral care.

A further, more recent Cochrane review identified 15 randomised controlled trials that improved oral care for people with stroke [17]. These trials ranged from education interventions to complex interventions including training, oral healthcare protocol, assessment and equipment [31]. Only two trials focussed on specialist training, one for registered nurses [32] and one for informal carers [29] in stroke. Across these trials training improved knowledge of oral care however, the quality of evidence was low. As highlighted in our study, further research could focus on staff training needs, increase protocol availability and the use of standardised assessment tools in practice.

### Strengths & limitations

The survey had a good response rate in both countries, which suggests that staff viewed oral care as an important topic and engaged with the study. However, the study used a self-reporting questionnaire which could have led to response and recall bias. Despite the respondents being encouraged to consult other team members, it is unclear to what extent this happened, thus responses may only reflect the respondent’s experience and may not be fully indicative of practice within their hospital.

## Conclusion

Our study findings have highlighted the considerable variability in oral care practices for stroke between the two countries. Oral care is a neglected area of stroke clinical practice, particularly in Australian hospitals. Oral care could be improved by increasing availability of training for staff, performing individual assessments on admission, and using standardised assessment tools and protocols to guide high quality care. Further research could focus on these possibilities and their incorporation into existing clinical practice.

## Supplementary Information

**Additional file 1.**

**Additional file 2: Table S1.** Likelihood of undertaking oral care assessments in the UK and in Australia. Categories unsure, not applicable and no response have been omitted. **Table S2.** Patient factors reported to influence whether an oral care assessment was undertaken for the UK and Australia. Categories unsure and no response have been omitted. **Table S3.** Staff factors, organisational factors and patient factors reported to influence oral care provision in the UK and Australia. Categories unsure and no response have been omitted. **Figure S1.** Oral care products available in UK (left) and Australian (right) hospitals.

## Data Availability

The datasets used and/or analysed during the current study are available from the corresponding author on reasonable request.
