# Supplementary material for: Oral care practices in stroke: findings from the UK and Australia
Source: BMC Nurs. 2021 Sep 15;20:169. doi: 10.1186/s12912-021-00642-y (PMC8442320; doi:10.1186/s12912-021-00642-y)
Supplement: Supplementary file 1 — Additional file 1. [file 12912_2021_642_MOESM1_ESM.docx]

Oral Health Care Practices

in Acute Stroke Care

# An international survey

| 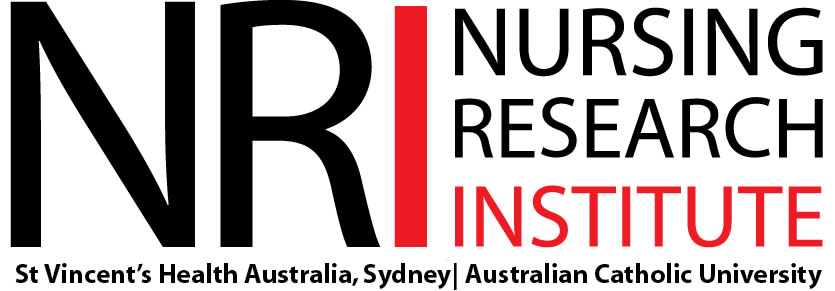 | 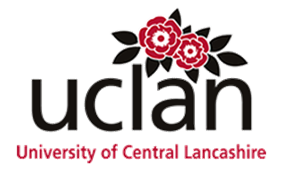 | 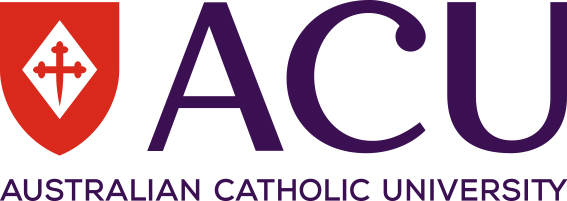 |
| --- | --- | --- |
| 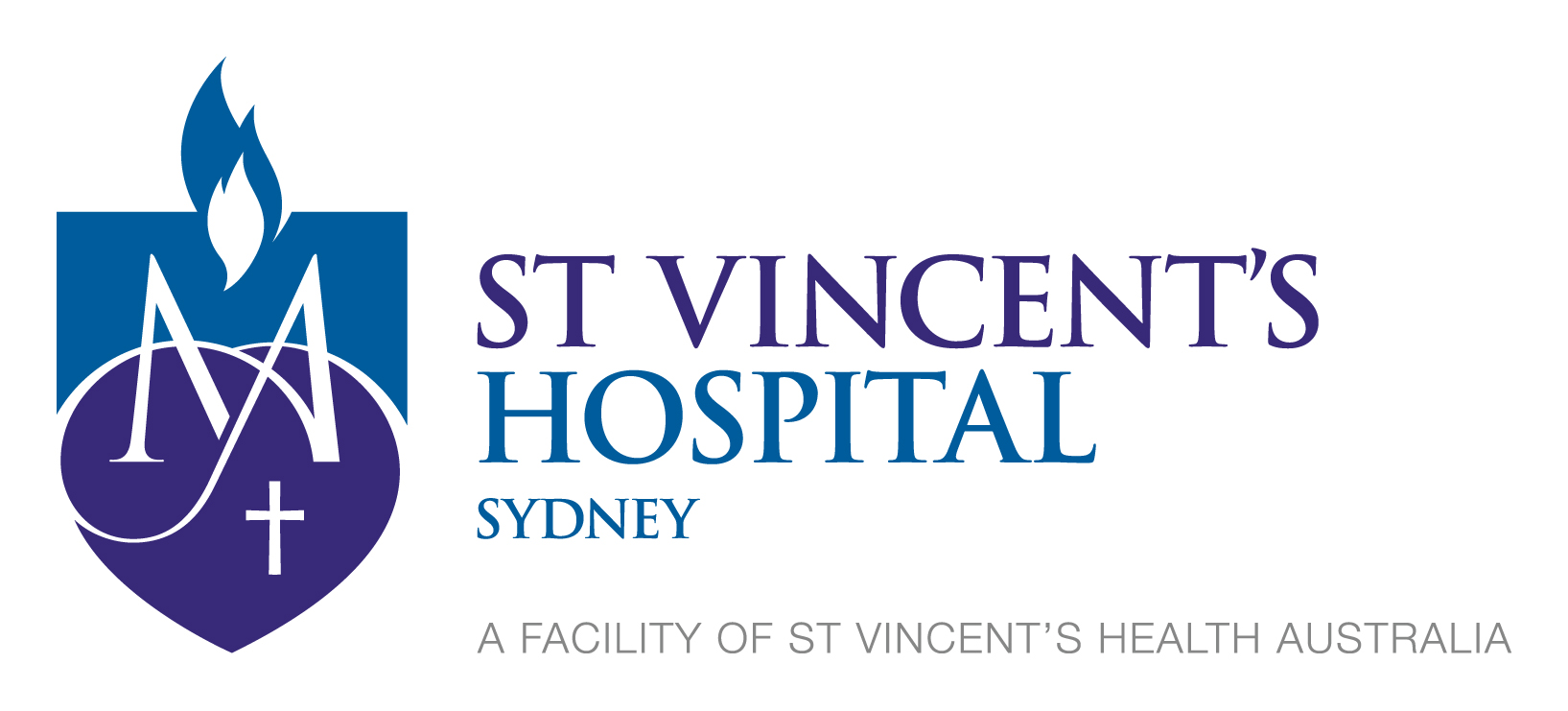 | 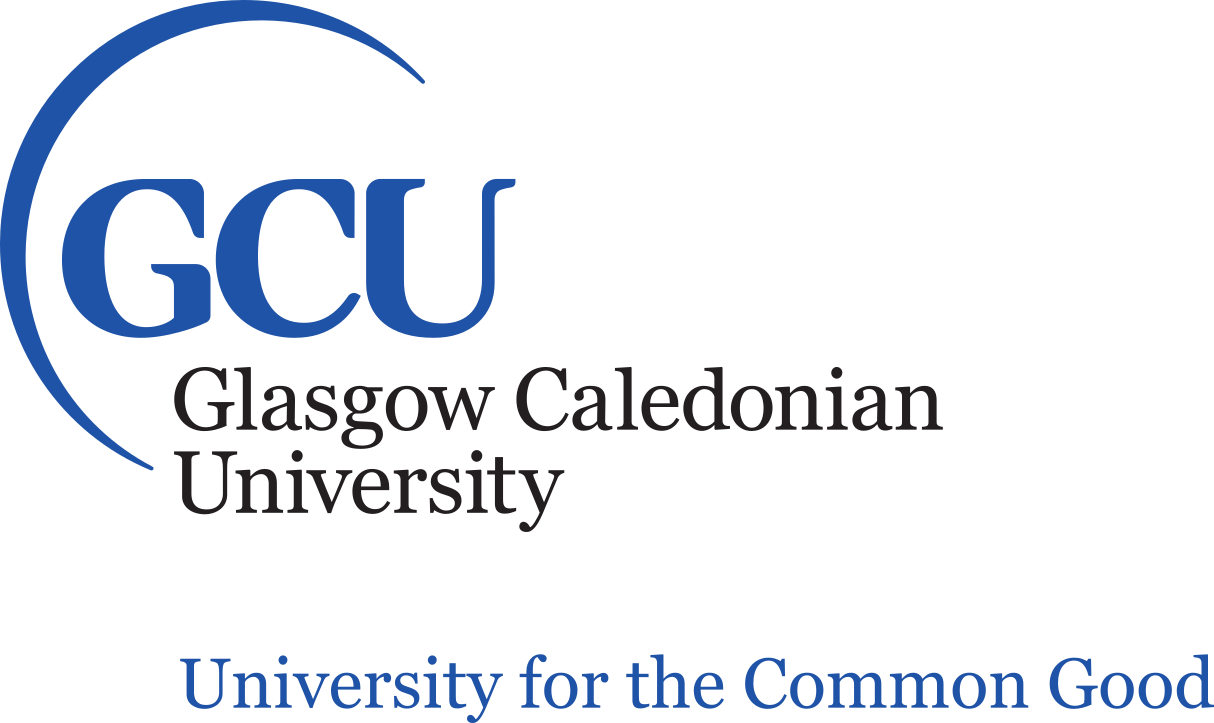 | 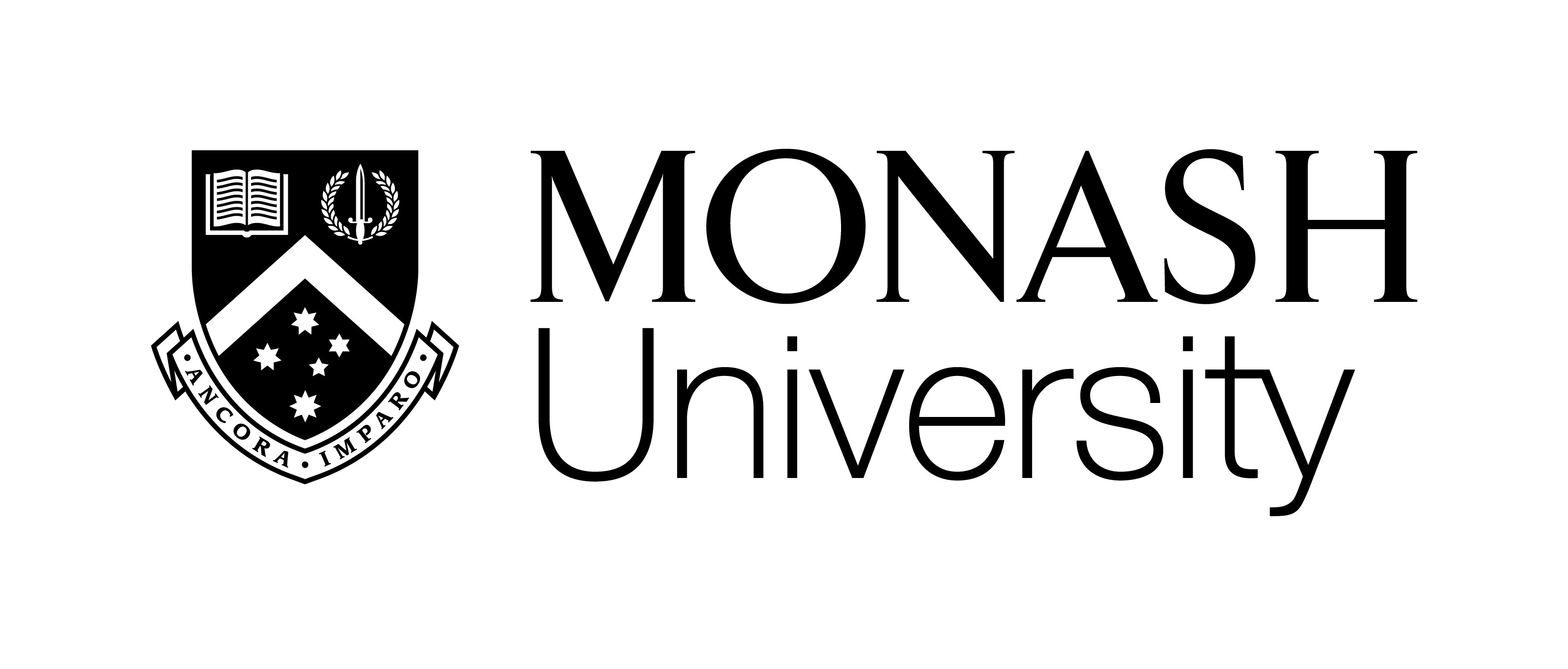 |

|  |
| --- |
| Thank you for taking part in this survey  **The questions in this survey refer to patients with stroke who are cared for in the stroke unit or in other wards.**  We encourage that you **consult** **with your colleagues** who provide oral health care for acute stroke patients **to assist in the completion of the questionnaire**.    The survey results will provide an understanding of current oral care practices for stroke patients in hospital. This information will help to identify barriers and enablers to oral health care, inform whether specific education for oral health care is required and identify topics for future research  Some answers require more than one response. Please read the questions and instructions carefully and respond as accurately as possible.  Thank you again for your participation |

**Section 1 Demographics and characteristics of person completing survey.**

### **Please provide your job title. *Please tick all that apply***

|  | Registered Nurse |  | Dentist |
| --- | --- | --- | --- |
|  | Clinical Nurse Specialist |  | Oral/ Maxillofacial Surgeon |
|  | Clinical Nurse Consultant |  | Consultant: Neurologist/ Geriatrician/ Physician |
|  | Nurse Unit Manager |  | Medical Registrar |
|  | Nurse Practitioner (stroke) |  | Speech and Language Therapist/ Speech Pathologist |
|  | Clinical Nurse Educator |  | Occupational Therapist |
|  | Stroke Liaison Nurse |  | Physician Assistant/Associate |
|  | Stroke Coordinator |  | Other, ***please specify***:_________________________ |

### **Do you have a stroke-specific role within your facility or service?**

If yes, tick one of the following:

|  | Stroke Coordinator |  | Stroke Unit Director | |
| --- | --- | --- | --- | --- |
|  | Stroke Liaison Nurse |  | |  |
|  | Clinical Nurse Consultant | | | |
|  | Other, ***please specify***: ________________________________________________ | | | |

If no, tick box

|  | No |
| --- | --- |

### **How long have you worked in your current role?**

|  | Years |  | Months |
| --- | --- | --- | --- |

### **What is your gender?**

|  | Male |  | Female |  | Other |
| --- | --- | --- | --- | --- | --- |

### **What is your age?**

|  | 21-30 years |
| --- | --- |
|  | 31-40 years |
|  | 41-50 years |
|  | 51-60 years |
|  | > 60 years |

### **Please indicate below the roles of all those who may have helped you to complete the survey.**

***Please tick all that apply.***

|  | Registered Nurse |  | Dentist |
| --- | --- | --- | --- |
|  | Clinical Nurse Specialist |  | Oral/ Maxillofacial Surgeon |
|  | Clinical Nurse Consultant |  | Consultant: Neurologist/ Geriatrician/ Physician |
|  | Nurse Unit Manager |  | Medical Registrar |
|  | Nurse Practitioner (stroke) |  | Speech and Language Therapist/ Speech Pathologist |
|  | Clinical Nurse Educator |  | Occupational Therapist |
|  | Stroke Liaison Nurse |  | Physician Assistant/Associate |
|  | Stroke Coordinator |  | Other, ***please specify***:_________________________ |

**7.** **What best describes the unit or ward where you work?**

|  | Acute stroke Unit |
| --- | --- |
|  | Ward with stroke beds, but not a ‘formal acute stroke unit’ |
|  | Integrated unit (acute and rehab beds in the same ward) |
|  | Rehabilitation unit |
|  | Other, ***please specify***:________________________________________________ |

**Section 2 Hospital and stroke service characteristics.**

### **What category below best describes your hospital setting?**

|  | Tertiary referral, University or Teaching Hospital |
| --- | --- |
|  | Non-tertiary, General, District or Community Hospital – with Emergency Department |
|  | Non-tertiary, General, District or Community Hospital – without Emergency Department |
|  | Other, ***please specify***:_______________________________________________ |

### **Please tell us about your stroke service:**

***(Please select one only)***

|  | We have a dedicated stroke unit with clinicians who have stroke expertise |
| --- | --- |
|  | We **do not** have a dedicated stroke unit, but ward(s) with stroke beds |
|  | We are a free-standing rehabilitation hospital *(go to question 11)* |

**9a. Which of the options below are provided by your acute stroke service?**

***(Please select all that apply)***

|  | *Service available* | *This is a*  *24/7 service* | |
| --- | --- | --- | --- |
|  |  | *Y* | *N* |
| Neurovascular imaging | *⃝* | *⃝* | *⃝* |
| Thrombolysis | *⃝* | *⃝* | *⃝* |
| Endovascular therapy | *⃝* | *⃝* | *⃝* |
| Neurosurgery | *⃝* | *⃝* | *⃝* |
| Telemedicine | *⃝* | *⃝* | *⃝* |
| Rehabilitation | *⃝* |  |  |

### **Please tell us about your rehabilitation service**

***(Please select one only)***

|  | Rehabilitation ward within acute hospital in **same** building of **same** health campus |
| --- | --- |
|  | Rehabilitation ward within acute hospital in **separate** buildings of **same** health campus |
|  | Rehabilitation ward within acute hospital in **separate** buildings on a **separate** health campus |
|  | Rehabilitation service within acute hospital (no dedicated beds, but ward(s) with stroke rehabilitation beds) |

**Section 3 Oral health care practices for patients with stroke.**

This section focuses on oral health care practices for patients with stroke in your stroke unit or ward. This section is **not** about swallow screening protocols.

### **Does your stroke unit or the ward where the majority of your patients with stroke are managed, have a protocol or guidelines about oral health care practices after acute stroke?**

|  | Yes – stroke patient specific oral care protocol |
| --- | --- |
|  | Yes – general oral care protocol for all patients |
|  | No *(go to question 13)* |
|  | Don’t know *(go to question 13)* |

### **How likely are clinical staff to use the oral care protocol?**

|  | Highly Likely |
| --- | --- |
|  | Likely |
|  | Unsure |
|  | Unlikely |
|  | Highly Unlikely |

### **Have staff working at your hospital received training in oral care provision in the last year?**

|  | Yes |
| --- | --- |
|  | No *(go to question 16)* |
|  | Unsure *(go to question 16)* |

### **Which staff groups working in stroke care have received training in oral care provision?**

***Please tick all that apply.***

|  | Registered Nurse | |
| --- | --- | --- |
|  | Enrolled Nurse | |
|  | Healthcare Assistant (UK)/ Assistant in Nursing (AUS) | |
|  | Physician Assistant/Associate | |
|  | Student Nurse | |
|  | Specialist Stroke Nurse (Clinical Nurse Consultant, Clinical Nurse Specialist, Nurse Practitioners) | |
|  | Speech and Language Therapist / Speech Pathologist | |
|  | Occupational Therapist | |
|  | Unsure | |
|  | Other, ***please specify***: | ______________________________________________________ |

### **Who provided the training?**

***Please tick all that apply.***

|  | Clinical Nurse Educator | |
| --- | --- | --- |
|  | Nurse Practitioner | |
|  | Speech therapist/pathologist | |
|  | Specialist Stroke Nurse (Clinical Nurse Consultant, Clinical Nurse Specialist, Nurse Practitioner) | |
|  | Dentist/ Dental Hygienist | |
|  | Other hospital dental staff | |
|  | Nurse educator | |
|  | Informal (from other staff) | |
|  | External Health Professional/Educator | |
|  | Unsure | |
|  | Other, ***please specify***: | ______________________________________________________ |

### **Are dental professionals employed by your hospital?**

|  | Yes |  |
| --- | --- | --- |
|  | No |  |
|  | Unsure |  |

### **Do dental professionals support staff on your stroke unit or ward?**

|  | Yes |
| --- | --- |
|  | No *(go to question 21)* |
|  | Unsure *(go to question 21)* |

### **If yes, what best describes the service that dental professionals provide on your stroke unit or ward?**

|  | Referral or on request |
| --- | --- |
|  | Regular dedicated sessions |
|  | Unsure |
|  | Other***, please specify***:________________________________________________________ |

### **How often do you get help from dental professionals?**

***Please tick all that apply.***

|  | Several times a week |  | Once a week |  |
| --- | --- | --- | --- | --- |
|  | Once a month |  | Once every few months |  |
|  | Other:___________________________________________ | | |  |

### **Which dental professionals provide this help?**

### ***Please tick all that apply.***

|  | Patient’s own dental practitioner |  | Maxillofacial staff |  |
| --- | --- | --- | --- | --- |
|  | Dental hospital staff |  | Dental hygienists |  |
|  | Community dentists |  | Don’t know |  |
|  | No dental professionals provided help | | |  |
|  | Other, **please specify**:___________________________________________ | | |  |

**Section 4 Assessment of oral health care practices for stroke patients.**

This section is about assessment and provision of oral care for stroke patients who are in your stroke unit or ward.

### **Does your ward or unit use an oral care assessment tool?**

***Please tick all that apply.***

|  | No tool used | |
| --- | --- | --- |
|  | The Holistic and Reliable Oral Assessment Tool (THROAT) | |
|  | Oral Health Assessment Tool (OHAT) | |
|  | Oral Assessment Tool (Eiler) | |
|  | Oral Cavity Assessment Tool (OCAT) | |
|  | Lockwood’s Oral Health Assessment Tool (LOHAT) | |
|  | Beck Oral Assessment Scale (BOAS) | |
|  | Oral Assessment and Intervention tool (OAIT) | |
|  | Geriatric Oral Health Assessment Scale (GOHAI) | |
|  | Brief Oral Health Status Examination (BOHSE) | |
|  | Oral Assessment Guide (OAG) | |
|  | Rattenbury, Mooney, Bowen Mouth Assessment Tool (RMBMAT) | |
|  | Mouth Care Assessment Tool (MCAT) | |
|  | Daily oral health assessment (DOHA) | |
|  | Local area/hospital specific tool | |
|  | Unsure |  |
|  | Other, ***please specify***:_____________________________________ | |

### **How likely would an oral care assessment be undertaken at the following times?**

***Please tick one box on each line.***

| **Frequency of assessment** | **Highly Likely** | **Likely** | **Unsure** | **Unlikely** | **Highly Unlikely** | **N/A** |
| --- | --- | --- | --- | --- | --- | --- |
| On admission to ward/unit |  |  |  |  |  |  |
| Every nursing shift |  |  |  |  |  |  |
| Daily |  |  |  |  |  |  |
| Weekly |  |  |  |  |  |  |
| As required or ad-hoc |  |  |  |  |  |  |
| On discharge |  |  |  |  |  |  |
| Other, ***please specify***:_____  _______________________  _______________________ |  |  |  |  |  |  |

### **How likely is each factor listed below to influence whether an oral care assessment is undertaken?**

***Please tick one box on each line.***

| **Patient factors** | **Highly Likely** | **Likely** | **Unsure** | **Unlikely** | **Highly Unlikely** |
| --- | --- | --- | --- | --- | --- |
| Dysphagia |  |  |  |  |  |
| Aphasia |  |  |  |  |  |
| Dysarthria |  |  |  |  |  |
| Cognitive impairment |  |  |  |  |  |
| Alert and able to self-manage |  |  |  |  |  |
| Unconsciousness |  |  |  |  |  |
| Physical impairment (upper limbs) |  |  |  |  |  |
| Physical impairment (lower limbs) |  |  |  |  |  |
| Nil by mouth |  |  |  |  |  |
| Inattention/visual field problems |  |  |  |  |  |
| Patient’s poor motivation |  |  |  |  |  |
| Malnourished |  |  |  |  |  |
| Dehydrated |  |  |  |  |  |
| Poor dental health |  |  |  |  |  |
| Own teeth |  |  |  |  |  |
| Dentures |  |  |  |  |  |
| Older age |  |  |  |  |  |
| Patient on medication that dries mouth |  |  |  |  |  |
| Facial weakness |  |  |  |  |  |
| Oxygen therapy |  |  |  |  |  |
| Other, ***please specify***  *_______________________________* |  |  |  |  |  |
| Other, ***please specify***  *_______________________________* |  |  |  |  |  |

### **How likely are the following professional groups to conduct an oral care assessment?**

***Please tick one box for each professional group.***

| **Professional group** | **Highly Likely** | **Likely** | **Unsure** | **Unlikely** | **Highly Unlikely** | **N/A** |
| --- | --- | --- | --- | --- | --- | --- |
| Registered Nurse |  |  |  |  |  |  |
| Enrolled Nurse/Advanced Diploma Nurse |  |  |  |  |  |  |
| Assistant in Nursing/Health care assistant |  |  |  |  |  |  |
| Student Nurse |  |  |  |  |  |  |
| Clinical Nurse Consultant |  |  |  |  |  |  |
| Specialist Stroke Nurse (including Clinical Nurse Consultant, Clinical Nurse Specialist, Nurse Practitioner) |  |  |  |  |  |  |
| Nurse Practitioner |  |  |  |  |  |  |
| Speech and Language Therapist/ Speech Pathologist |  |  |  |  |  |  |
| Occupational Therapist |  |  |  |  |  |  |
| Dieticians |  |  |  |  |  |  |
| Doctor |  |  |  |  |  |  |
| Dentist/ Dental Hygienist |  |  |  |  |  |  |
| Oral/ Maxillofacial Surgeon |  |  |  |  |  |  |
| Other, ***please specify***  *__________________________* |  |  |  |  |  |  |
| Other, ***please specify***  *__________________________* |  |  |  |  |  |  |

### **If a patient with stroke is incapable of independent oral care, how likely is each professional group to provide oral care?**

***Please tick one box for each professional group.***

| **Professional group** | **Highly Likely** | **Likely** | **Unsure** | **Unlikely** | **Highly Unlikely** | **N/A** |
| --- | --- | --- | --- | --- | --- | --- |
| Registered Nurse |  |  |  |  |  |  |
| Enrolled Nurse/Advanced Diploma Nurse |  |  |  |  |  |  |
| Assistant in Nursing/Health care assistant |  |  |  |  |  |  |
| Student Nurse |  |  |  |  |  |  |
| Clinical Nurse Consultant |  |  |  |  |  |  |
| Specialist Stroke Nurse (Clinical Nurse Consultant, Clinical Nurse Specialist, Nurse Practitioner) |  |  |  |  |  |  |
| Nurse Practitioner |  |  |  |  |  |  |
| Speech and Language Therapist/ Speech Pathologist |  |  |  |  |  |  |
| Occupational Therapist |  |  |  |  |  |  |
| Dieticians |  |  |  |  |  |  |
| Doctor |  |  |  |  |  |  |
| Dentist/ Dental Hygienist |  |  |  |  |  |  |
| Oral/ Maxillofacial Surgeon |  |  |  |  |  |  |
| Other***, please specify***:  ___________________________ |  |  |  |  |  |  |

### **If a patient is incapable of independent oral care, are family or carers (non-professionals) encouraged to provide oral care to patients with acute stroke?**

|  | Yes |
| --- | --- |
|  | No *(go to question 28)* |
|  | Unsure *(go to question 28)* |

### **If yes, do they receive any oral care training or guidance from health professionals?**

|  | Yes |
| --- | --- |
|  | No |
|  | Unsure |

### **If a patient with stroke is incapable of independent oral care, how often are staff expected to perform the cleaning of natural teeth, dentures and oral soft tissue?**

***Please tick one option for each of the three columns.***

| **Option** | **Cleaning of natural teeth** | **Cleaning of dentures** | **Cleaning of soft tissue** |
| --- | --- | --- | --- |
| Three times a day |  |  |  |
| Twice a day |  |  |  |
| Once a day |  |  |  |
| Weekly |  |  |  |
| Never |  |  |  |
| Unsure |  |  |  |
| Other, **please specify**:  _______________________ |  |  |  |

### **For patients incapable of independent oral care and who fall into any of the three groups in the table below, how often are staff expected to perform oral care for them?**

***Please tick one option for each of the three columns.***

| **Option** | **1. Nil by mouth** | **2. Modified diet** | **3. Normal diet** |
| --- | --- | --- | --- |
| Three times a day |  |  |  |
| Twice a day |  |  |  |
| Once a day |  |  |  |
| Weekly |  |  |  |
| Never |  |  |  |
| Unsure |  |  |  |
| Other, ***please specify***:  _______________________ |  |  |  |
|  |  |  |  |

### **If a patient can attend to their own oral care, how likely are they to be routinely assessed by staff on their ability to continue to perform their own oral care?**

|  | Highly likely |
| --- | --- |
|  | Likely |
|  | Unsure |
|  | Unlikely |
|  | Highly unlikely |

### **Are oral health care practices documented in each patient’s medical records?**

|  | Yes |
| --- | --- |
|  | No *(go to question 35)* |
|  | Unsure *(go to question 35)* |

### **Where are oral health care practices documented and how likely are they to be documented?**

***Please tick one box for each document type.***

| **Document type** | **Highly Likely** | **Likely** | **Unsure** | **Unlikely** | **Highly Unlikely** |
| --- | --- | --- | --- | --- | --- |
| Dedicated oral care form |  |  |  |  |  |
| Patient clinical/progress notes |  |  |  |  |  |
| Care plan |  |  |  |  |  |
| Clinical pathway |  |  |  |  |  |
| Observation chart |  |  |  |  |  |
| Other, ***please specify***:  ______________________________ |  |  |  |  |  |
| Other, ***please specify***:  ______________________________ |  |  |  |  |  |

### **How often are staff expected to document oral health care practices?**

***Please tick one box for each frequency of documentation option.***

| **Frequency of documentation** | **Never** | **Rarely** | **Sometimes** | **Often** | **Always** |
| --- | --- | --- | --- | --- | --- |
| On admission to ward/unit |  |  |  |  |  |
| Every nursing shift |  |  |  |  |  |
| Daily |  |  |  |  |  |
| Weekly |  |  |  |  |  |
| As required or ad-hoc |  |  |  |  |  |
| On discharge |  |  |  |  |  |
| Never |  |  |  |  |  |
| Other, ***please specify***:  _______________________________ |  |  |  |  |  |

### **What aspects of oral health care are documented and how likely are they to be documented?**

### ***Please tick one box for each oral care element.***

| **Oral care elements** | **Highly Likely** | **Likely** | **Unsure** | **Unlikely** | **Highly Unlikely** |
| --- | --- | --- | --- | --- | --- |
| Date/time oral care occurred |  |  |  |  |  |
| Frequency of oral care (e.g. how often attended) |  |  |  |  |  |
| Areas of the mouth cleaned (e.g. teeth, dentures etc.) |  |  |  |  |  |
| Equipment used to perform oral care |  |  |  |  |  |
| Who provided care (e.g. hospital staff; patient; carer) |  |  |  |  |  |
| Whether an oral health care plan was developed |  |  |  |  |  |
| All elements as above using a generic tick box option on a care plan or pathway that indicates ‘all oral health assessment and care attended’ |  |  |  |  |  |
| Other, ***please specify***:  _________________________________ |  |  |  |  |  |

**Section 5 Provision of oral health care resources.**

This section is about the equipment and resources available on your unit or ward to facilitate and support oral care for patients with stroke.

### **If patients do not have their own oral hygiene products, which of the following are provided on your stroke ward or unit?**

### ***Please tick all that apply.***

|  | Manual toothbrush | |  | Denture brush |
| --- | --- | --- | --- | --- |
|  | Electric toothbrush | |  | Steradent |
|  | Toothpaste | |  | Corsodyl/chlorhexidine |
|  | Foam swab | |  | Mouthwash tablets |
|  | Glycerine swab | |  | Sodium bicarbonate |
|  | Bleach | |  | Saline/sodium chloride solution |
|  | Mouthwash | |  | Sodium hypochlorite (Milton) |
|  | Dental floss | |  | Denture adhesive |
|  | Suction equipment | |  | Ascorbic acid/Vitamin C |
|  | Soft cloth/towel | |  | Other decontaminants e.g. Antibiotic gel |
|  | Tongue scraper, | |  | Other, ***please specify***:________________ |
|  | Suction toothbrush |  |  |  |

### **Which of the following are available for patients with a dry mouth?**

### ***Please tick all that apply.***

|  | Artificial saliva |  | Malic acid |
| --- | --- | --- | --- |
|  | Citric acid |  | Chewing gum |
|  | Lemon & glycerine swab |  | Nicotinamide/Vitamin B_3_ |
|  | Ascorbic acid/Vitamin C |  | Pilocarpine |
|  | Pastilles/lollipops |  | Biotene |
|  | Oral fluid (water/tea/soft drink) |  | None |
|  | Other, ***please specify****:___________________________________________________* | | |

**Section 6 Factors influencing oral care provision.**

This section focuses on your views about potential barriers to providing oral care to stroke patients.

### **Consider each statement below and indicate your level of agreement for each.**

|  | **Statement** | **Strongly agree** | **Agree** | **Unsure** | **Disagree** | **Strongly disagree** |
| --- | --- | --- | --- | --- | --- | --- |
| **Staff Factors** | Staff do not routinely document when oral care has been delivered to patients |  |  |  |  |  |
|  | I am satisfied with the level of oral care provided to patients in my ward/unit |  |  |  |  |  |
|  | Ward staff are too busy with other ward duties to conduct oral care |  |  |  |  |  |
|  | Staff shortages impact on staff capacity to deliver oral care |  |  |  |  |  |
|  | Nurses lack confidence in delivering oral health care |  |  |  |  |  |
|  | Oral care is perceived by nurses as less important than other aspects of patient care |  |  |  |  |  |
|  | Nurses lack awareness about the health benefits of oral health |  |  |  |  |  |
|  | There is a lack of evidence to support oral health care after stroke |  |  |  |  |  |
|  | I am happy with the level of oral health education provided on my ward/unit |  |  |  |  |  |
| **Organisational factors** | There is variability and inconsistency in oral health care provision |  |  |  |  |  |
|  | No assessment tool is used on my unit/ward to guide oral care assessment |  |  |  |  |  |
|  | Carers/family members are encouraged to undertake oral health care |  |  |  |  |  |
|  | Oral care after stroke is a neglected area of practice |  |  |  |  |  |
|  | Safety issues are a concern for staff and patients when undertaking oral health care i.e. aspiration |  |  |  |  |  |
|  | There is a lack of hospital and/or ward protocols on oral health care for patients after stroke |  |  |  |  |  |
|  | There is a lack of equipment i.e. toothbrushes, mouth rinses, dental floss, suction, on my unit/ward |  |  |  |  |  |
|  | There is a lack of access to specialist dental care at my hospital |  |  |  |  |  |
|  | Pre-registration education and training of nurses in oral health care provision is inadequate |  |  |  |  |  |
|  | Post-registration education and training of nurses in oral health care is inadequate |  |  |  |  |  |
| **Patient factors** | Difficulties communicating with stroke patients when attempting to deliver oral care is a barrier |  |  |  |  |  |
|  | Altered patient sensory perception is a barrier to oral care, i.e. hypersensitivity, pain, numbness |  |  |  |  |  |
|  | Stroke patients may have an altered sensation of thirst |  |  |  |  |  |
|  | It is difficult to provide oral care after stroke to patients with cognitive impairment |  |  |  |  |  |
