# Supplementary material for: Oral care practices in stroke: findings from the UK and Australia
Source: BMC Nurs. 2021 Sep 15;20:169. doi: 10.1186/s12912-021-00642-y (PMC8442320; doi:10.1186/s12912-021-00642-y)

Supplementary material

Supplement Table 1. Likelihood of undertaking oral care assessments in the UK and in Australia. Categories unsure, not applicable and no response have been omitted.

|  | **UK n (%)** | | **Australia n (%)** | |
| --- | --- | --- | --- | --- |
| Frequency of assessment | Likely | Unlikely | Likely | Unlikely |
| On admission to ward/unit | 109 (73) | 24 (16) | 68 (57) | 31 (26) |
| Every nursing shift | 83 (55) | 41 (27) | 38 (32) | 45 (38) |
| Daily | 97 (65) | 26 (17) | 57 (48) | 29 (24) |
| Weekly | 82 (55) | 23 (15) | 51 (43) | 23 (19) |
| As required or ad-hoc | 82 (55) | 17 (11) | 81 (68) | 9 (7.5) |
| On discharge | 26 (17) | 71 (47) | 13 (11) | 57 (48) |

Supplement Table 2. Patient factors reported to influence whether an oral care assessment was undertaken for the UK and Australia. Categories unsure and no response have been omitted.

|  | **UK n (%)** | | **Australia n (%)** | |
| --- | --- | --- | --- | --- |
| Patient factors | Likely | Unlikely | Likely | Unlikely |
| Unconsciousness | 136 (91) | 2 (1.3) | 102 (85) | 4 (3.3) |
| Cognitive impairment | 116 (77) | 15 (10) | 65 (54) | 27 (23) |
| Dysphagia | 139 (93) | 2 (1.3) | 108 (90) | 7 (5.8) |
| Facial weakness | 117 (78) | 14 (9.3) | 84 (70) | 15 (13) |
| Inattention/visual field problems | 96 (64) | 26 (17) | 39 (33) | 40 (33) |
| Physical impairment (upper limbs) | 113 (75) | 19 (13) | 63 (53) | 29 (24) |
| Physical impairment (lower limbs) | 62 (41) | 59 (39) | 20 (17) | 70 (58) |
| Aphasia | 98 (65) | 27 (18) | 80 (67) | 18 (15) |
| Dysarthria | 103 (69) | 22 (15) | 85 (71) | 14 (12) |
| Dehydrated | 131 (87) | 7 (4.7) | 79 (66) | 20 (17) |
| Malnourished | 120 (80) | 11 (7.3) | 77 (64) | 26 (22) |
| Poor dental health | 133 (89) | 8 (5.3) | 84 (70) | 19 (16) |
| Own teeth | 89 (59) | 32 (21) | 44 (37) | 48 (40) |
| Dentures | 117 (78) | 14 (9.3) | 71 (59) | 26 (22) |
| Patient’s poor motivation | 101 (67) | 21 (14) | 47 (39) | 41 (34) |
| Older age | 99 (66) | 19 (13) | 59 (49) | 31 (26) |
| Alert and able to self-manage | 49 (33) | 85 (57) | 22 (18) | 83 (69) |
| Nil by mouth | 143 (95) | 2 (1.3) | 100 (83) | 6 (5.0) |
| Oxygen therapy | 127 (85) | 10 (6.7) | 52 (43) | 37 (31) |
| Patient on medication that dries mouth | 109 (73) | 18 (12) | 59 (49) | 31 (26) |

Supplement Table 3: Staff factors, organisational factors and patient factors reported to influence oral care provision in the UK and Australia. Categories unsure and no response have been omitted.

|  | **UK n (%)** | | **Australia n (%)** | |
| --- | --- | --- | --- | --- |
|  | **Agree** | **Disagree** | **Agree** | **Disagree** |
| **Staff factors** |  |  |  |  |
| Staff shortages impact on staff capacity to deliver oral care. | 96 (64) | 47 (31) | 67 (56) | 38 (32) |
| Ward staff are too busy with other ward duties to conduct oral care. | 52 (35) | 87 (58) | 47 (39) | 50 (42) |
| Staff do not routinely document when oral care has been delivered to patients. | 63 (42) | 81 (54) | 91 (76) | 26 (22) |
| There is a lack of evidence to support oral health care after stroke. | 38 (25) | 88 (59) | 17 (14) | 74 (62) |
| Nurses lack confidence in delivering oral health care. | 40 (27) | 86 (57) | 25 (21) | 74 (62) |
| Oral care is perceived by nurses as less important than other aspects of patient care. | 49 (33) | 86 (57) | 59 (49) | 44 (37) |
| Nurses lack awareness about the health benefits of oral health. | 61 (41) | 76 (51) | 59 (49) | 48 (40) |
| I am satisfied with the level of oral care provided to patients in my ward/unit. | 64 (43) | 63 (42) | 38 (32) | 71 (59) |
| I am happy with the level of oral health education provided on my ward/unit. | 54 (36) | 78 (52) | 22 (18) | 82 (68) |
|  |  |  |  |  |
| **Organisational factors** |  |  |  |  |
| Oral care after stroke is a neglected area of practice | 73 (49) | 62 (41) | 74 (62) | 28 (23) |
| There is a lack of hospital and/or ward protocols on oral health care for patients after stroke. | 93 (62) | 43 (29) | 96 (80) | 13 (11) |
| No assessment tool is used on my unit/ward to guide oral care assessment. | 75 (50) | 69 (46) | 102 (85) | 12 (10) |
| There is a lack of equipment i.e. toothbrushes, mouth rinses, dental floss, suction, on my unit/ward. | 36 (34) | 105 (70) | 35 (29) | 79 (66) |
| Pre-registration education and training of nurses in oral health care provision is inadequate. | 95 (63) | 23 (15) | 64 (53) | 15 (13) |
| Post-registration education and training of nurses in oral health care is inadequate. | 95 (63) | 35 (23) | 77 (64) | 14 (12) |
| There is variability and inconsistency in oral health care provision. | 102 (68) | 31 (21) | 89 (74) | 12 (10) |
| Safety issues are a concern for staff and patients when undertaking oral health care i.e. aspiration. | 76 (51) | 52 (35) | 50 (42) | 44 (37) |
| There is a lack of access to specialist dental care at my hospital. | 106 (71) | 30 (20) | 87 (73) | 20 (17) |
| Carers/family members are encouraged to undertake oral health care. | 103 (69) | 38 (25) | 78 (65) | 29 (24) |
|  |  |  |  |  |
| **Patient factors** |  |  |  |  |
| It is difficult to provide oral care after stroke to patients with cognitive impairment. | 93 (62) | 49 (33) | 80 (67) | 28 (23) |
| Altered patient sensory perception is a barrier to oral care, i.e. hypersensitivity, pain, numbness. | 98 (65) | 36 (24) | 60 (50) | 44 (37) |
| Difficulties communicating with stroke patients when attempting to deliver oral care is a barrier | 87 (58) | 54 (36) | 64 (53) | 43 (36) |
| Stroke patients may have an altered sensation of thirst. | 103 (69) | 15 (10) | 82 (68) | 11 (9.2) |

Supplement Figure 1: Oral care products available in UK (left) and Australian (right) hospitals


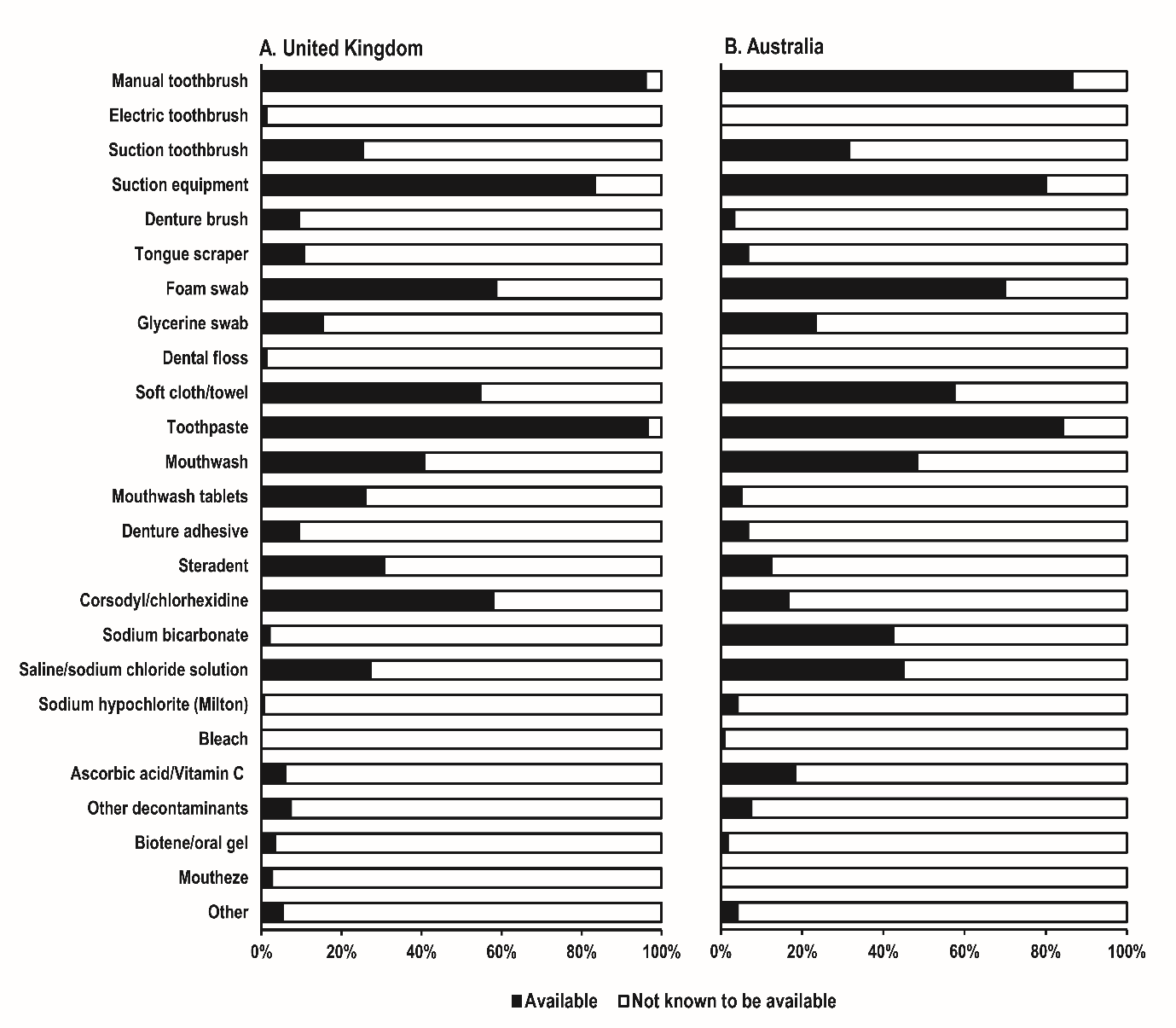

Supplement: Supplementary file 2 — Additional file 2: Table S1. Likelihood of undertaking oral care assessments in the UK and in Australia. Categories unsure, not applicable and no response have been omitted. Table S2. Patient factors reported to influence whether an oral care assessment was undertaken for the UK and Australia. Categories unsure and no response have been omitted. Table S3. Staff factors, organisational factors and patient factors reported to influence oral care provision in the UK and Australia. Categories unsure and no response have been omitted. Figure S1. Oral care products available in UK (left) and Australian (right) hospitals. [file 12912_2021_642_MOESM2_ESM.docx]
